# Single-Nucleotide Polymorphisms (SNPs) Both Associated with Hypertension and Contributing to Accelerated-Senescence Traits in OXYS Rats

**DOI:** 10.3390/ijms21103542

**Published:** 2020-05-17

**Authors:** Vasiliy A. Devyatkin, Olga E. Redina, Natalia A. Muraleva, Nataliya G. Kolosova

**Affiliations:** 1 Institute of Cytology and Genetics, Siberian Branch of Russian Academy of Sciences (ICG SB RAS), 10 Lavrentyeva Ave., Novosibirsk 630090, Russia; devyatkinvasiliy@gmail.com (V.A.D.); oredina@bionet.nsc.ru (O.E.R.); kolosova@bionet.nsc.ru (N.G.K.); 2 Novosibirsk State University, 2 Pirogova Str., Novosibirsk 630090, Russia

**Keywords:** aging, age-related disease, hypertension, SNP, senescence-accelerated OXYS rat

## Abstract

Aging is a major risk factor of numerous human diseases. Adverse genetic variants may contribute to multiple manifestations of aging and increase the number of comorbid conditions. There is evidence of links between hypertension and age-related diseases, although the genetic relationships are insufficiently studied. Here, we investigated the contribution of hypertension to the development of accelerated-senescence syndrome in OXYS rats. We compared transcriptome sequences of the prefrontal cortex, hippocampus, and retina of OXYS rats with the genotypes of 45 rat strains and substrains (which include models with hypertension) to find single-nucleotide polymorphisms (SNPs) both associated with hypertension and possibly contributing to the development of age-related diseases. A total of 725 polymorphisms were common between OXYS rats and one or more hypertensive rat strains/substrains being analyzed. Multidimensional scaling detected significant similarities between OXYS and ISIAH rat genotypes and significant differences between these strains and the other hypertensive rat strains/substrains. Nonetheless, similar sets of SNPs produce a different phenotype in OXYS and ISIAH rats depending on hypertension severity. We identified 13 SNPs causing nonsynonymous amino-acid substitutions having a deleterious effect on the structure or function of the corresponding proteins and four SNPs leading to functionally significant structural rearrangements of transcripts in OXYS rats. Among them, SNPs in genes *Ephx1*, *Pla2r1*, and *Ccdc28b* were identified as candidates responsible for the concomitant manifestation of hypertension and signs of accelerated aging in OXYS rats.

## 1. Introduction

Aging is a risk factor for many age-related diseases, but their risk depends on genetic factors, environmental conditions, lifestyle, and the presence of other pathologies. The prevalence of age-related diseases is growing against the background of an unprecedented aging rate of the world’s population. The situation is complicated by the fact that over 60% of people aged over 65 are affected by multiple diseases, which are, therefore, more difficult to treat. Adverse genetic variations may accelerate aging in adults, thereby contributing to premature morbidity, disability, and/or mortality. More systematic investigation into the mechanisms of aging leading to multiple diseases is needed to identify the key nodes to target. There is strong evidence of the links between many age-related diseases and age-associated vascular dysfunction, although no causal relationships are yet identified. Naturally, hypertension is one of the risk factors of many age-related diseases, and it affects 7% of adults <40 years of age, where the prevalence increases with aging, reaching two-thirds of people over 60 years old [1]. Destructive effects of hypertension on the circulatory system—e.g., aneurysms, a reduction in blood vessel elasticity, and impaired heart function leading to hypoperfusion and metabolic stress—are regarded by many authors as risk factors not only for cardiovascular morbidity and mortality [2] but also for a cognitive decline and neurodegenerative changes [3]. According to data from a meta-analysis and several individual studies, hypertension is a moderate risk factor of age-related macular degeneration (AMD) [4]. Recently, the significant genetic heterogeneity of Alzheimer’s disease (AD) was revealed among subjects with and without hypertension [5]. Nevertheless, the causal relationships of hypertension with other age-related disorders remain unknown, and this state of affairs is related to the genetic heterogeneity of the majority of age-related diseases. A promising way to clarify this causation is to study animal models, whose genetic uniformity and standard conditions of maintenance reduce the influence of external factors and increase the reproducibility of research results.

Here, we investigated the contribution of hypertension to the development of accelerated-senescence syndrome in OXYS rats (ICG SB RAS); this syndrome is characterized by early manifestation of a phenotype similar to human geriatric disorders [6,7,8]. Against the background of moderately high blood pressure, OXYS rats spontaneously develop cataract, hypertrophic cardiomyopathy, sarcopenia, osteoporosis, AMD-like retinopathy, and AD-like pathology in the absence of mutations in genes (*App*, *Psen1*, and *Psen2*) that are specific for the familial form of AD [9,10]. The OXYS rat strain was created via selection for susceptibility to the cataractogenic effect of a galactose-rich diet in the first five generations from Wistar/Icgn rats. After selection for early spontaneous cataracts, subsequent generations of the rats without the galactose-rich diet acquired the accelerated-senescence phenotype. Currently, we have the 116th generation of OXYS rats with spontaneously developing cataract and accelerated-senescence syndrome, which means early development of a phenotype similar to a human geriatric disorder accompanied by moderate hypertension. Recently, in the genome of OXYS rats, we uncovered some single-nucleotide polymorphisms (SNPs) that may affect the manifestation of traits of the accelerated-senescence phenotype in these rats, with a focus on AD-like and AMD-like pathologies [11] and mitochondrial dysfunction [12]. The purpose of the present study was to search for SNPs both associated with moderate arterial hypertension and possibly contributing to the development of age-related diseases in OXYS rats.

## 2. Results

### 2.1. Genetic Similarities of the Rat Strains and Substrains

A comparison of transcriptome sequences of the prefrontal cortex, hippocampus, and retina of OXYS rats with the reference genome of BN/NHsdMcwi rats revealed 42,478 SNPs in 9903 genes. The list of these SNPs was compared with the genotypes of the other 45 rat strains and substrains, including 12 strains and substrains of hypertensive rats (FHH/EurMcwi, LH/MavRrrc, MHS/Gib, SBH/Ygl, SHR/OlaIpcv, SHRSP/Gla, SHR/NCrlPrin, SHR/NHsd, SHR/OlaIpcvPrin, SS/Jr, SS/JrHsdMcwi, and ISIAH/Icgn), 11 strains/substrains that commonly serve as a normotensive control (FHL/EurMcwi, LN/MavRrrc, LL/MavRrrc, MNS/Gib, SBN/Ygl, SR/Jr, WKY/N, WKY/Gla, WKY/NCrl, WKY/NHsd, and WAG/GSto-Icgn), and 22 rat strains/substrains often used as a control or experimental group in the studies on various pathological conditions unrelated to hypertension or aging (ACI/N, ACI/EurMcwi, BBDP/Wor, BN-Lx/Cub, BN-Lx/CubPrin, BN/SsN, BUF/N, DA/BklArbNsi, F334/N, F344/NHsd, F344/NCrl, SUO_F344, GK/Ox, LE/Stm [SOLiD], LEW/Crl, LEW/NCrlBR, LE/Stm [Illumina], M520/N, MR/N, WAG/Rij, WN/N, and Wistar/Icgn).

**Figure 1:**
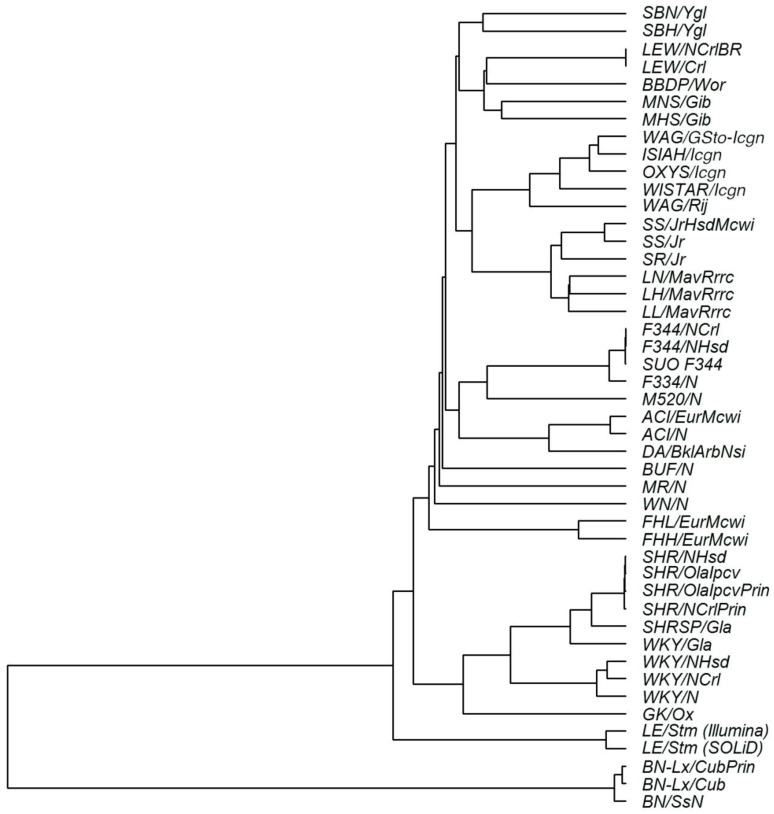
Analysis of genetic similarities of rat strains and substrains in terms of single-nucleotide polymorphisms (SNPs). The dendrogram is based on the identity of alleles by state. SNPs were identified in the transcriptomes of three tissues of OXYS/Icgn rats and in the corresponding loci of the WAG/GSto-Icgn, ISIAH/Icgn, and Wistar/Icgn rat transcriptomes or genomes available in the databases for the other 42 rat strains and substrains.

Analysis of the genetic similarity based on the identified SNPs showed that OXYS/Icgn rats are genetically closer to the ISIAH/Icgn rat strain (which was also derived from Wistar/Icgn rats) and to two strains of Wistar Albino Glaxo rats (WAG/GSto-Icgn and WAG/Rij) than to the other 41 strains and substrains being studied (Figure 1).

**Figure 2:**
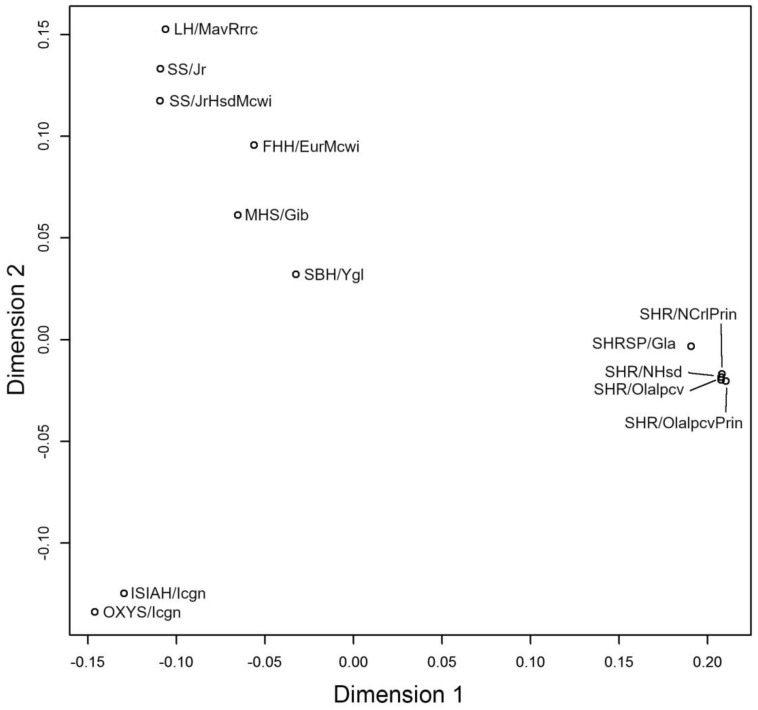
Multidimensional-scaling analysis of distances (allele identity by state) between the genotype of OXYS/Icgn rats and genotypes of 12 other hypertensive rat strains and substrains.

Multidimensional scaling was performed to visualize similarities between the genotypes of OXYS rats and 12 other strains/substrains of hypertensive rats (Figure 2). This analysis uncovered substantial similarity of genotypes between OXYS and ISIAH rats and substantial differences of these two strains from the rest of the hypertensive rat strains and substrains. Figure 2 also indicates that rats with salt-sensitive hypertension (SS/Jr, SS/JrHsdMcwi, and SBH/Ygl) and rat strains developing spontaneous hypertension (SHR/OlaIpcv, SHR/NCrlPrin, SHR/NHsd, SHR/OlaIpcvPrin, and SHRSP/Gla) are genetically distant from each other.

### 2.2. SNPs Detected in Both OXYS Rats and in One or More Other Hypertensive Strains/Substrains

**Figure 3:**
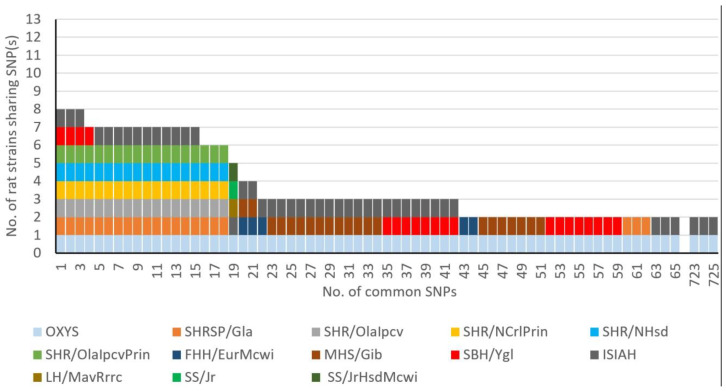
Numbers of common SNPs between OXYS rats and hypertensive rat strains. The colors correspond to the SNPs that were identified in one of the strains of hypertensive rats being analyzed.

Among the 42,478 SNPs found in OXYS rats, 40,373 SNPs in 9699 genes were also detected in the genomes of normotensive rat strains or substrains unrelated to hypertension or age-related diseases. Among the other 2105 SNPs, 725 polymorphisms were found in both OXYS rats and in one or more hypertensive rat strains/substrains under study (Table S1).The numbers of common nucleotide variants among the genotypes of hypertensive strains and substrains are presented in Figure 3. Most of these SNPs (663 out of 725) were common between OXYS/Icgn and ISIAH/Icgn rats and were not found in other hypertensive rat strains/substrains. Additionally, Figure 3 clearly indicates that no SNP was common for all the 13 hypertensive rat strains/substrains under study. The highest frequency of an SNP among the hypertensive rat strains/substrains was eight out of 13 strains, and four out of these eight strains were represented by SHR substrains. This result highlights differences in the genetic basis of different forms of hypertension in rats.

The most common SNPs (found in at least six hypertensive rat strains/substrains being analyzed) are listed in Table 1. These nucleotide substitutions were found in the messenger RNAs (mRNAs) of 14 genes, two of which (*Pla2r1* and *Yars*) are known to be associated with mental disorders and neurodegenerative diseases, respectively. None of the genes presented in Table 1 were associated with hypertension to date. The SNPs (in *Pla2r1* and *Ccdc28b*) causing nonsynonymous amino-acid substitutions can lead to changes in the structure and/or function of the respective proteins. Most of the nucleotide substitutions presented in Table 1 are located in noncoding regions of the genes.

Classification of the effects of the 725 SNPs that can influence the hypertensive status of OXYS rats is shown in Table 2. Four SNPs were classified as “exerting a high impact on transcript structure” (Table 3). Two of these were found in the mRNA sequence of *Csnk1e*, which is annotated in the Rat Genome Database (RGD) as a gene associated with neurodegenerative diseases and mental disorders. One of these substitutions (c.1521A > G) leading to a loss of a stop codon is common between OXYS and hypertensive SBH/Ygl rats, and the other substitution (c.1083G > A), which creates a stop codon, is common between OXYS and two other hypertensive rat strains (SBH/Ygl and ISIAH/Icgn; Table S1).

The SIFT algorithm detected 13 SNPs presumably having a significant negative effect on the structure and/or function of a protein (Table 4). Only one gene (*Ephx1*) from those listed in Table 4 is currently known to be associated with hypertension. In addition, *Ephx1* and several other genes (*Pla2r1*, *Zmym6*, *Trappc9*, and *Nqo2*) are annotated in the RGD as genes associated with neurodegenerative diseases and/or mental disorders (Table 4). SNPs in two of the genes (*Pla2r1* and *Ccdc28b*) listed in Table 4 are among the most common among the hypertensive rats; SNPs in these genes were detected both in OXYS rats and in six other hypertensive strains/substrains. We then performed functional annotation of the genes containing the SNPs common between OXYS rats and one or more hypertensive rat strains. The results revealed enrichment with such functional categories as GTP/ATP binding, various signaling systems, and cell division.

## 3. Discussion

In this study, special attention was given to a comparative analysis of transcriptomic sequence data from OXYS rats and sequences available for 12 other hypertensive rat strains/substrains that model different forms of hypertension. No SNPs were found that are present in all the 13 hypertensive strains/substrains under study. Common SNPs were detected in no more than eight out of the 13 analyzed hypertensive strains/substrains. This result is consistent with evidence that hypertension is a genetically heterogeneous disorder, and that this conclusion may be true for humans (it was obtained by the analysis of several strains/substrains of rats with different mechanisms behind the development of hypertension). Because nonsynonymous amino-acid substitutions can affect protein function, it is believed that they have the greatest impact on human health [13]. We identified 13 SNPs causing nonsynonymous amino-acid substitutions having a deleterious effect on the structure or function of the corresponding protein and four SNPs leading to functionally significant structural rearrangements of transcripts in OXYS rats. We believe that these 17 nucleotide substitutions most likely include SNPs both associated with moderate arterial hypertension and contributing to age-related diseases in OXYS rats. These 17 SNPs belong to a set of genes where only one (*Ephx1*) is currently associated with hypertension. Furthermore, *Ephx1* correlates with neurodegenerative diseases and mental disorders and accordingly can be considered one of the most likely candidate genes responsible for the relation between elevated blood pressure and the signs of neurodegeneration during aging.

In our work, the RGD was employed to identify the genes associated with hypertension. On the other hand, according to the results presented in Reference [14], *Gtpbp4* (GTP-binding protein 4) is a candidate gene associated with hypertension in three SHR rat substrains. On the basis of this information, it can be assumed that a nonsynonymous substitution presumably affecting the structure and/or function of GTPBP4 may be associated with hypertension in both OXYS and ISIAH rats.

To identify other candidate SNPs that may both be implicated in moderate arterial hypertension and contribute to age-related diseases in OXYS rats, we focused primarily on those that occur in at least several strains of hypertensive rats. Thus, we propose that the nucleotide substitutions that were found in the mRNAs of the 14 genes presented in Table 1 are of interest for further research on their contribution to the development of hypertension in several rat strains. Two of these genes, *Pla2r1* and *Ccdc28b*, can be regarded as promising candidate genes; they contain nucleotide substitutions that are believed to alter the function or structure of the protein and were found both in OXYS rats and in six other hypertensive rat strains and substrains.

Both genes are located in the genetic loci associated with blood pressure in the above-mentioned rat strains. In the region of chromosome 3 where the *Pla2r1* gene is located, BpQTLcluster4 (blood pressure QTL cluster 4) was found in SHR rats [15] and a quantitative trait locus, Bp118 (blood pressure QTL 118), was identified in SHRSP rats [16]. *Ccdc28b* is located in the region of chromosome 5 where the genetic loci associated with blood pressure were mapped in studies on SHR rats: Bp103 (blood pressure QTL 103) [17] and Bp139 (blood pressure QTL 139) [18]. So far, genes *Pla2r1* and *Ccdc28b* are not yet associated with hypertension; however, according to our results, they hold promise for further research into their role in the hypertensive state in OXYS rats and in many other rat strains modeling hypertension.

Several SNPs around the *Pla2r1* gene (phospholipase A2 receptor 1) are reported to be significantly associated with idiopathic membranous nephropathy [19], which is the most common cause of nephrotic syndrome and renal failure [20]. Detection of high PLA2R1 serum titers, which has high sensitivity and specificity for idiopathic membranous nephropathy, was also reported in a study on a patient with type 1 diabetes, diabetic retinopathy, arterial hypertension, and nephrotic syndrome [21]. Renal histological examination of this patient revealed extensive glomerular and vascular sclerotic changes attributable to diabetes and hypertension [21]. A knockout of *Pla2r1* in a mouse model of progeria attenuates some premature-aging signs, such as rib fracture and decreased bone content, while simultaneously decreasing a senescence marker level [22].

The protein encoded by the *Ccdc28b* gene (coiled coil domain-containing 28B) is involved in ciliogenesis and exerts a modifier effect on Bardet–Biedl syndrome [23,24]. This syndrome is an autosomal recessive disorder, and its characteristic features include obesity, cognitive impairment, tapetoretinal degeneration, mental retardation, renal disorders, and hypertension [25,26].

Based on these data, it can be hypothesized that the SNPs found in the *Pla2r1* and *Ccdc28b* mRNA sequences (these SNPs are present in the genotypes of several hypertensive rat strains but not found in normotensive rats) may be interesting in terms of the research into their effects on the development of hypertension, both in model animals and in humans. In addition, the *Pla2r1* gene is known to be associated with mental disorders, and *Ccdc28b* is related to retinal dystrophy. Accordingly, we can theorize that these two genes are highly probable contributors both to the hypertensive state and to the signs of accelerated aging in OXYS rats.

It is also worth mentioning that 11 of the 14 genes presented in Table 1 are located in the same genetic region of chromosome 5 within a relatively small locus (from 147.4 to 147.9 megabases). Keeping in mind that the SNP in the *Ccdc28b* gene is one of the most promising polymorphisms localized in this region of chromosome 5, we can suggest that this genomic region may contain one or several other genes responsible for the hypertensive state of rat strains that model different forms of hypertension. 

Most of the nucleotide substitutions listed in Table 1 are located in noncoding regions. As demonstrated in a number of studies, SNPs in the regulatory regions of mRNA [27,28,29,30] and in introns [27,28,29,30,31,32] can have a substantial modifying effect on the processes of transcription and translation, thereby often leading to various pathologies. In accordance with existing knowledge about the possible contribution of this kind of SNPs to the development of pathologies, it is likely that nucleotide substitutions that we found in the noncoding regions of transcripts in several strains of hypertensive rats may be important in this regard and could be interesting for further study of their role in increasing arterial blood pressure in several hypertensive rat strains, including OXYS rats.

It is worth mentioning that the SHRSP/Gla strain is among the hypertensive strains in which SNPs were found in *Pla2r1* and *Ccdc28b*. These rats with spontaneous hypertension and a high risk of stroke are characterized by spontaneously developing cerebrovascular disorders [33]. The features of stroke in SHRSP rats are believed to be similar to the clinical manifestations of this disease in humans [34]. In this regard, we reported previously that cerebrovascular dysfunction, neurovascular alterations (including accumulation of amyloid β), an impairment of cerebral blood flow, increased neuronal degeneration, and susceptibility to hypoxia and ischemia contribute to the development of AD-like pathology in OXYS rats [35].

Our present data revealed numerous common polymorphisms between the transcriptomes of OXYS and ISIAH rats. This phenomenon can be explained by the fact that both OXYS and ISIAH rats were selected from the same Wistar/Icgn rat stock in the 1970s, but the selection was carried out by means of different traits. As mentioned earlier, the selection of OXYS rats was carried out on the signs of early spontaneous cataract. Genetically linked with the latter was a set of features of accelerated senescence that include moderate hypertension. ISIAH rats were selected on the basis of an increased reaction of blood pressure to restriction stress [36]. It is known that hypertension can result in hypertrophic cardiomyopathy. Despite the differences in hypertension severity, both rat strains—ISIAH [37] and OXYS [38]—are known to develop hypertrophic cardiomyopathy. At the same time, in ISIAH rats, there are no other manifestations of accelerated senescence that are typical for OXYS rats, including signs of accelerated brain aging. In particular, the two strains differ significantly in behavioral stereotypes. The accelerated aging in OXYS rats is associated with a reduction in locomotor and exploratory activities and in learning, as well as with memory deficits against a background of the emergence of other key signs of AD. On the contrary, ISIAH rats are characterized by increased exploratory activity and are less anxious than control normotensive rats [39]. Nonetheless, our findings suggest that hypertension pathogenesis in OXYS rats may be similar to that in ISIAH/Icgn rats, which have a large number of common SNPs with OXYS rats.

Thus, the results of this study mean that different forms of hypertension have specific genetic contributing factors. To identify the SNPs both associated with moderate arterial hypertension and possibly contributing to age-related diseases in OXYS rats, we primarily considered the SNPs that are present in the largest number of hypertensive strains and can cause changes of protein structure and/or function. The SNPs of genes *Ephx1*, *Pla2r1*, and *Ccdc28b* were found to be candidates for the simultaneous manifestation of elevated blood pressure and signs of the accelerated-senescence phenotype in OXYS rats. These SNPs can be considered good candidates for the involvement in the development of hypertension and premature aging in humans. In addition, we found that, among the dozens of analyzed strains, ISIAH rats are genetically closest to OXYS rats, and this finding may be applied as a filter to identify the SNPs specific for the OXYS rat strain alone and possibly contributing to its accelerated-senescence phenotype.

## 4. Materials and Methods

### 4.1. Animals

We used 20-day-old and three-, five-, and 18-month-old male senescence-accelerated OXYS rats, whereas age-matched male Wistar rats served as controls (3–5 per group). The animals were maintained at the Center for Genetic Resources of Laboratory Animals at the ICG SB RAS under standard laboratory conditions (22 ± 2 °C, 60% relative humidity, 12-h light/12-h dark cycle, and lights on at 9:00 a.m.). Feed and water were available ad libitum. The protocol of the animal experiment was approved by the Bioethical Committee of the ICG SB RAS, according to The Guidelines for Manipulations with Experimental Animals (the decree of the Presidium of the Russian Academy of Sciences No. 12000-496 of 2 April 1980).

### 4.2. Tissue Preparation

The prefrontal cortex, retina, and hippocampus were excised rapidly after decapitation, placed in RNAlater (Ambion Inc., Austin, TX, USA, catalog # AM7020), frozen, and stored at −20 °C until analysis. Total RNA was isolated using the TRIzol Reagent (Invitrogen, Carlsbad, CA, USA, cat. # 15596-018), and RNA quality and quantity were evaluated on an Agilent Bioanalyzer (Agilent Technologies, Santa Clara, CA, USA).

### 4.3. High-Throughput RNA Sequencing (RNA-seq)

We analyzed RNA-seq data obtained in previous studies for each sample of retinal, prefrontal cortex, and hippocampal RNA [7,35]. RNA-seq was performed at Genoanalytica Inc. (www.genoanalytica.ru, Moscow, Russia) in accordance with standard Illumina protocols (mRNA-Seq Sample Prep Kit) by Illumina nonstranded sequencing (on an Illumina GA IIx at Genoanalytica) as described previously [7,35]. Reading depth reached 50 million reads, and all samples were analyzed as biological replicates for each rat group.

### 4.4. SNP Detection

After barcode trimming, the quality of the sequencing data was tested in the FastQC software (https://www.bioinformatics.babraham.ac.uk/projects/fastqc/). We used Bowtie 2 (http://bowtie-bio.sourceforge.net/bowtie2/index.shtml) and TopHat software v2.0.4 (https://ccb.jhu.edu/software/tophat/index.shtml) for mapping to *Rattus norvegicus* reference genome assembly RGSC 6.0 (Ensemble release 75) and the CollectRnaSeqMetrics module in the Picard software package (https://broadinstitute.github.io/picard/) for the estimation of mapping quality.

SNP positions within the aligned reads in relation to the reference genome were identified by means of SAMtools (v. 0.1.17) (http://www.htslib.org/). The obtained polymorphic variants with minimum mapping quality (Q) of 100 in the tissues of OXYS rats were next filtered by custom Java scripts according to the following criteria: only those positions were selected whose genotype was found to be in a homozygous state for at least three animals in one experimental group. Depth of coverage (DP) had to be ≥ 10 in at least one animal.

The list of SNPs of OXYS rats was next compared with RGSC 6.0 data for genome sequences of 45 rat strains and substrains: 11 strains/substrains used as a normotensive control (FHL/EurMcwi, LN/MavRrrc, LL/MavRrrc, MNS/Gib, SBN/Ygl, SR/Jr, WKY/N, WKY/Gla, WKY/NCrl, WKY/NHsd, and WAG/GSto-Icgn), 22 rat strains/substrains with a phenotype not related to hypertension or aging (ACI/N, ACI/EurMcwi, BBDP/Wor, BN-Lx/Cub, BN-Lx/CubPrin, BN/SsN, BUF/N, DA/BklArbNsi, F334/N, F344/NHsd, F344/NCrl, SUO_F344, GK/Ox, LE/Stm [SOLiD], LEW/Crl, LEW/NCrlBR, LE/Stm [Illumina], M520/N, MR/N, WAG/Rij, WN/N, and Wistar), and 12 strains and substrains manifesting signs of hypertension (FHH/EurMcwi, LH/MavRrrc, MHS/Gib, SBH/Ygl, SHR/OlaIpcv, SHRSP/Gla, SHR/NCrlPrin, SHR/NHsd, SHR/OlaIpcvPrin, SS/Jr, SS/JrHsdMcwi, and ISIAH/Icgn) [40]. A comparison of OXYS rat SNPs with genomes of the 45 rat strains and substrains was performed only at genomic loci sequenced during this transcriptome analysis of OXYS rats.

### 4.5. Prediction of SNP Phenotypic Effects and Functional Annotation

We employed the Variant Effect Predictor (https://www.ensembl.org/Multi/Tools/VEP) tool to determine the influence of amino-acid substitutions on protein function; the consequence type and SIFT score were predicted for each variant. SIFT scores of 0–0.05 were assumed to mean a “deleterious” effect, and scores of 0.05–1.00 were regarded as a “tolerated effect.” The gene list with SNPs was subjected to functional enrichment analyses by means of the DAVID tool (https://david.ncifcrf.gov/). The RGD (https://rgd.mcw.edu) was used to identify associations of SNP-containing genes with various phenotypes.

### 4.6. The Dendrogram

The identity by state (IBS) analysis of the alleles was carried out using SNPRelate in the R software environment [41] to measure the distances between objects for dendrogram construction and for demonstrating the results on principal coordinates.

## Figures and Tables

**Table 1:** SNPs that are most common among the hypertensive rat strains/substrains under study. Chr.—chromosome; ID—identifier.

Gene Symbol	Chr.: Position	ID	Classification	SNP	Amino-Acid Substitution	Rat Strains
*Pla2r1* ^∆^	3: 46,597,362	rs198261397	missense_variant	c.574G > A	Glu192Lys^^^	SHR/OlaIpcv, SHRSP/Gla, SHR/NCrlPrin, SHR/NHsd, SHR/OlaIpcvPrin, SBH/Ygl, OXYS/Icgn
*AC132627.2*	5: 147,674,509	rs197330026	upstream_gene_variant	-	-	SHR/OlaIpcv, SHRSP/Gla, SHR/NCrlPrin, SHR/NHsd, SHR/OlaIpcvPrin, ISIAH/Icgn, OXYS/Icgn
*Bsdc1*	5: 147,674,509	rs197330026	synonymous_variant	c.618A > C	Ala206Ala	SHR/OlaIpcv, SHRSP/Gla, SHR/NCrlPrin, SHR/NHsd, SHR/OlaIpcvPrin, ISIAH/Icgn, OXYS/Icgn
	5: 147,685,455	rs198904367	intron_variant	c.1148-981A > G	-	SHR/OlaIpcv, SHRSP/Gla, SHR/NCrlPrin, SHR/NHsd, SHR/OlaIpcvPrin, OXYS/Icgn
	5: 147,688,443	rs198439815	3_prime_UTR_variant	c.*57A > C	-	SHR/OlaIpcv, SHRSP/Gla, SHR/NCrlPrin, SHR/NHsd, SHR/OlaIpcvPrin, ISIAH/Icgn, OXYS/Icgn
	5: 147,688,529	rs198616603	3_prime_UTR_variant	c.*143C > T	-	SHR/OlaIpcv, SHRSP/Gla, SHR/NCrlPrin, SHR/NHsd, SHR/OlaIpcvPrin, ISIAH/Icgn, OXYS/Icgn
	5: 147,688,798	rs197462955	3_prime_UTR_variant	c.*412C > T	-	SHR/OlaIpcv, SHRSP/Gla, SHR/NCrlPrin, SHR/NHsd, SHR/OlaIpcvPrin, ISIAH/Icgn, OXYS/Icgn
	5: 147,688,823	rs198095674	3_prime_UTR_variant	c.*437C > T	-	SHR/OlaIpcv, SHRSP/Gla, SHR/NCrlPrin, SHR/NHsd, SHR/OlaIpcvPrin, ISIAH/Icgn, OXYS/Icgn
	5: 147,688,857	rs198680609	3_prime_UTR_variant	c.*471G > A	-	SHR/OlaIpcv, SHRSP/Gla, SHR/NCrlPrin, SHR/NHsd, SHR/OlaIpcvPrin, ISIAH/Icgn, OXYS/Icgn
*Ccdc28b*	5: 147,824,835	-	missense_variant	c.499C > T	Arg167Cys^^^	SHR/OlaIpcv, SHRSP/Gla, SHR/NCrlPrin, SHR/NHsd, SHR/OlaIpcvPrin, ISIAH/Icgn, OXYS/Icgn
	5: 147,827,114	-	intron_variant	c.164 + 134C > T	-	SHR/OlaIpcv, SHRSP/Gla, SHR/NCrlPrin, SHR/NHsd, SHR/OlaIpcvPrin, OXYS/Icgn
*Fam229a*	5: 147,688,443	rs198439815	upstream_gene_variant	-	-	SHR/OlaIpcv, SHRSP/Gla, SHR/NCrlPrin, SHR/NHsd, SHR/OlaIpcvPrin, ISIAH/Icgn, OXYS/Icgn
	5: 147,688,529	rs198616603	upstream_gene_variant	-	-	SHR/OlaIpcv, SHRSP/Gla, SHR/NCrlPrin, SHR/NHsd, SHR/OlaIpcvPrin, ISIAH/Icgn, OXYS/Icgn
	5: 147,688,798	rs197462955	upstream_gene_variant	-	-	SHR/OlaIpcv, SHRSP/Gla, SHR/NCrlPrin, SHR/NHsd, SHR/OlaIpcvPrin, ISIAH/Icgn, OXYS/Icgn
	5: 147,688,823	rs198095674	upstream_gene_variant	-	-	SHR/OlaIpcv, SHRSP/Gla, SHR/NCrlPrin, SHR/NHsd, SHR/OlaIpcvPrin, ISIAH/Icgn, OXYS/Icgn
	5: 147,688,857	rs198680609	upstream_gene_variant	-	-	SHR/OlaIpcv, SHRSP/Gla, SHR/NCrlPrin, SHR/NHsd, SHR/OlaIpcvPrin, ISIAH/Icgn, OXYS/Icgn
*Iqcc*	5: 147,824,835	-	upstream_gene_variant	-	-	SHR/OlaIpcv, SHRSP/Gla, SHR/NCrlPrin, SHR/NHsd, SHR/OlaIpcvPrin, ISIAH/Icgn, OXYS/Icgn
	5: 147,827,114	-	upstream_gene_variant	-	-	SHR/OlaIpcv, SHRSP/Gla, SHR/NCrlPrin, SHR/NHsd, SHR/OlaIpcvPrin, OXYS/Icgn
*Kpna6*	5: 147,852,765	-	3_prime_UTR_variant	c.*478G > A	-	SHR/OlaIpcv, SHRSP/Gla, SHR/NCrlPrin, SHR/NHsd, SHR/OlaIpcvPrin, ISIAH/Icgn, OXYS/Icgn
*Rbbp4*	5: 147,508,661	rs197189353	synonymous_variant	c.873G > A	Thr291Thr	SHR/OlaIpcv, SHRSP/Gla, SHR/NCrlPrin, SHR/NHsd, SHR/OlaIpcvPrin, ISIAH/Icgn, OXYS/Icgn
*RGD1561149*	5: 147,407,313	rs198649532	missense_variant	c.2327C > T	Ser776Leu	SHR/OlaIpcv, SHRSP/Gla, SHR/NCrlPrin, SHR/NHsd, SHR/OlaIpcvPrin, ISIAH/Icgn, OXYS/Icgn
*Tssk3*	5: 147,685,455	rs198904367	downstream_gene_variant	-	-	SHR/OlaIpcv, SHRSP/Gla, SHR/NCrlPrin, SHR/NHsd, SHR/OlaIpcvPrin, OXYS/Icgn
	5: 147,688,443	rs198439815	downstream_gene_variant	-	-	SHR/OlaIpcv, SHRSP/Gla, SHR/NCrlPrin, SHR/NHsd, SHR/OlaIpcvPrin, ISIAH/Icgn, OXYS/Icgn
	5: 147,688,529	rs198616603	downstream_gene_variant	-	-	SHR/OlaIpcv, SHRSP/Gla, SHR/NCrlPrin, SHR/NHsd, SHR/OlaIpcvPrin, ISIAH/Icgn, OXYS/Icgn
	5: 147,688,798	rs197462955	downstream_gene_variant	-	-	SHR/OlaIpcv, SHRSP/Gla, SHR/NCrlPrin, SHR/NHsd, SHR/OlaIpcvPrin, ISIAH/Icgn, OXYS/Icgn
	5: 147,688,823	rs198095674	downstream_gene_variant	-	-	SHR/OlaIpcv, SHRSP/Gla, SHR/NCrlPrin, SHR/NHsd, SHR/OlaIpcvPrin, ISIAH/Icgn, OXYS/Icgn
	5: 147,688,857	rs198680609	downstream_gene_variant	-	-	SHR/OlaIpcv, SHRSP/Gla, SHR/NCrlPrin, SHR/NHsd, SHR/OlaIpcvPrin, ISIAH/Icgn, OXYS/Icgn
*Yars* ^#^	5: 147,407,313	rs198649532	downstream_gene_variant	-	-	SHR/OlaIpcv, SHRSP/Gla, SHR/NCrlPrin, SHR/NHsd, SHR/OlaIpcvPrin, ISIAH/Icgn, OXYS/Icgn
*Zbtb8os*	5: 147,541,343	rs106772412	synonymous_variant	c.144C > G	Thr48Thr	SHR/OlaIpcv, SHRSP/Gla, SHR/NCrlPrin, SHR/NHsd, SHR/OlaIpcvPrin, ISIAH/Icgn, OXYS/Icgn
*Tmem132c*	12: 33,016,103	rs198544729	synonymous_variant	c.783G > C	Gly261Gly	SHR/OlaIpcv, SHRSP/Gla, SHR/NCrlPrin, SHR/NHsd, SHR/OlaIpcvPrin, OXYS/Icgn
*Fech*	18: 59,942,888	rs13449838	3_prime_UTR_variant	c.*702G > A	-	SHR/OlaIpcv, SHRSP/Gla, SHR/NCrlPrin, SHR/NHsd, SHR/OlaIpcvPrin, SBH/Ygl, ISIAH/Icgn, OXYS/Icgn
	18: 59,943,052	rs199183859	3_prime_UTR_variant	c.*538G > A	-	SHR/OlaIpcv, SHRSP/Gla, SHR/NCrlPrin, SHR/NHsd, SHR/OlaIpcvPrin, SBH/Ygl, ISIAH/Icgn, OXYS/Icgn
	18: 59,943,291	rs13449883	3_prime_UTR_variant	c.*299A > G	-	SHR/OlaIpcv, SHRSP/Gla, SHR/NCrlPrin, SHR/NHsd, SHR/OlaIpcvPrin, SBH/Ygl, ISIAH/Icgn, OXYS/Icgn

^^^ Mutations that presumably have a significant (deleterious) effect on the structure and/or function of the corresponding protein according to the SIFT algorithm; * untranslated region; ^#^ neurodegenerative diseases; ^∆^ mental disorders. - polymorphisms that do not alter the sequence of the coding part or amino-acid residues.

**Table 2:** The classification of SNP effects that can influence the accelerated-aging phenotype in OXYS rats.

Nucleotide Substitution Effect	Impact	Number of SNPs
frameshift_variant	High	4
stop_gained	High	4
stop_lost	High	1
synonymous_variant	Low	338
splice_region_variant, synonymous_variant	Low	5
synonymous_variant, NMD_transcript_variant	Low	1
splice_region_variant, 3_prime_UTR_variant, NMD_transcript_variant	Low	1
missense_variant	Moderate	159
missense_variant, NMD_transcript_variant	Moderate	4
missense_variant, splice_region_variant	Moderate	4
intergenic_variant	Modifier	243
3_prime_UTR_variant	Modifier	533
intron_variant	Modifier	751
downstream_gene_variant	Modifier	1262
upstream_gene_variant	Modifier	414
5_prime_UTR_variant	Modifier	75
non_coding_transcript_exon_variant	Modifier	49
intron_variant, non_coding_transcript_variant	Modifier	38
intron_variant, NMD_transcript_variant	Modifier	12
3_prime_UTR_variant, NMD_transcript_variant	Modifier	5

NMD: nonsense-mediated messenger RNA (mRNA) decay, UTR: untranslated region.

**Table 3:** SNPs causing functionally significant structural rearrangements of transcripts that may be related to the development of the hypertensive state in OXYS rats.

Chr.	Position	Gene Symbol	Gene Definition	Transcript	Classification	Substitution	Hypertensive Rat Strains/Substrains
7	120,652,704	*Csnk1e* ^# ∆^	Casein kinase 1, epsilon	ENSRNOT00000018126	stop_lost	c.1521A > G	SBH/Ygl
7	120,658,002			ENSRNOT00000087800	stop_gained	c.1083G > A	SBH/Ygl, ISIAH
20	6,556,093	*Lemd2*	LEM domain-containing protein 2-like	ENSRNOT00000035819	frameshift_variant	c.141dup	ISIAH
	46,519,455	*AABR07045405.1*		ENSRNOT00000077765	frameshift_variant	c.29del	ISIAH

Genes associated with ^#^ neurodegenerative diseases (including Alzheimer’s disease (AD)) or ^∆^ mental disorders (including neurocognitive disorders).

**Table 4:** Mutations that presumably have a significant influence on the structure and/or function of the respective protein according to the SIFT algorithm and may be related to the development of the hypertensive state in OXYS rats.

Chr.	Position	SNP_ID	Gene Symbol	Gene Name	Transcript	Substitution	Amino-Acid Change	Hypertensive Rat Strains/Substrains
3	46,597,362	rs198261397	*Pla2r1* ^∆^	Phospholipase A2 receptor 1	ENSRNOT00000079261	c.355G > A	Glu119Lys	SBH/Ygl, SHR/OlaIpcv, SHRSP/Gla, SHR/NCrlPrin, SHR/NHsd, SHR/OlaIpcvPrin
					ENSRNOT00000011003	c.574G > A	Glu192Lys	SBH/Ygl, SHR/OlaIpcv, SHRSP/Gla, SHR/NCrlPrin, SHR/NHsd, SHR/OlaIpcvPrin
3	164,232,288	-	*Spata2*	Spermatogenesis-associated 2	ENSRNOT00000012604	c.632G > A	Arg211Gln	ISIAH
5	145,185,401	-	*Zmym6* ^#^	Zinc finger MYM-type-containing 6	ENSRNOT00000019135	c.2983C > T	Arg995Cys	ISIAH
					ENSRNOT00000079732	c.3100C > T	Arg1034Cys	ISIAH
5	147,824,835	-	*Ccdc28b*	Coiled coil domain-containing 28B	ENSRNOT00000075659	c.499C > T	Arg167Cys	SHR/OlaIpcv, SHRSP/Gla, SHR/NCrlPrin, SHR/NHsd, SHR/OlaIpcvPrin, ISIAH
					ENSRNOT00000083369			SHR/OlaIpcv, SHRSP/Gla, SHR/NCrlPrin, SHR/NHsd, SHR/OlaIpcvPrin, ISIAH
7	77,988,396	-	*Slc25a32*	Mitochondrial folate transporter/carrier solute carrier family 25 member 32	ENSRNOT00000006029	c.947G > T	Thr316Ile	ISIAH
7	113,986,760	-	*Trappc9* ^∆^	Trafficking protein particle complex 9	ENSRNOT00000038172	c.3397C > T	Arg1133Cys	MHS/Gib, ISIAH
7	117,497,524	-	*Mroh1*	Maestro heat-like repeat family member 1	ENSRNOT00000081632	c.3022C > T	Arg1008Cys	ISIAH
7	117,723,996	-	*Kifc2*	Kinesin family member C2	ENSRNOT00000085152	c.307G > A	Gly103Arg	ISIAH
					ENSRNOT00000093128			ISIAH
13	99,281,863	-	Ephx1* ^# ∆^	Epoxide hydrolase 1	ENSRNOT00000004780	c.296A > T	Asn99Ile	ISIAH
					ENSRNOT00000085279			ISIAH
17	32,136,113	-	*Nqo2* ^# ∆^	*N*-Ribosyl-dihydronicotinamide:quinone reductase 2	ENSRNOT00000024141	c.370G > A	Gly124Arg	ISIAH
17	63,123,745	-	*Gtpbp4*	GTP-binding protein 4	ENSRNOT00000074389	c.905A > G	Glu302Gly	ISIAH
20	5,414,829	-	*RT1-A1*	RT1 class Ia, locus A1	ENSRNOT00000041590	c.124C > T	Arg42Trp	ISIAH
					ENSRNOT00000078972	c.133C > T	Arg45Trp	ISIAH
					ENSRNOT00000080900			ISIAH
20	47,395,226	-	*Ostm1*	Osteoclastogenesis-associated transmembrane protein 1	ENSRNOT00000057116	c.182T > C	Leu61Ser	ISIAH

Genes associated with * hypertension, ^#^ neurodegenerative diseases, or ^∆^ mental disorders.

## Supplementary Materials

Supplementary materials can be found at https://www.mdpi.com/1422-0067/21/10/3542/s1. Table S1. Rats and one or more hypertensive strains/substrains.

## References

[1] Ong K.L., Cheung B.M., Man Y.B., Lau C.P., Lam K.S. (2007). Prevalence awareness treatment and control of hypertension among United States adults 1999–2004. Hypertension.

[2] Kjeldsen S.E. (2018). Hypertension and cardiovascular risk: General aspects. Pharmacol. Res..

[3] Csiszar A., Tarantini S., Fülöp G.A., Kiss T., Valcarcel-Ares M.N., Galvan V., Ungvari Z., Yabluchanskiy A. (2017). Hypertension impairs neurovascular coupling and promotes microvascular injury: Role in exacerbation of Alzheimer’s disease. Geroscience.

[4] Lipecz A., Miller L., Kovacs I., Czakó C., Csipo T., Baffi J., Csiszar A., Tarantini S., Ungvari Z., Yabluchanskiy A. (2019). Microvascular contributions to age-related macular degeneration (AMD): From mechanisms of choriocapillaris aging to novel interventions. Geroscience.

[5] Nazarian A., Arbeev K.G., Yashkin A.P., Kulminski A.M. (2019). Genetic heterogeneity of Alzheimer’s disease in subjects with and without hypertension. Geroscience.

[6] Kolosova N.G., Stefanova N.A., Korbolina E.E., Fursova A.Z., Kozhevnikova O.S. (2014). Senescence-accelerated OXYS rats: A genetic model of premature aging and age-related diseases. Adv. Gerontol..

[7] Kozhevnikova O.S., Korbolina E.E., Ershov N.I., Kolosova N.G. (2013). Rat retinal transcriptome: Effects of aging and AMD-like retinopathy. Cell Cycle.

[8] Muraleva N.A., Ofitserov E.N., Tikhonov V.P., Kolosova N.G. (2012). Efficacy of glucosamine alendronate alone & in combination with dihydroquercetin for treatment of osteoporosis in animal model. Indian J. Med. Res..

[9] Stefanova N.A., Maksimova K.Y., Kiseleva E., Rudnitskaya E.A., Muraleva N.A., Kolosova N.G. (2015). Melatonin attenuates impairments of structural hippocampal neuroplasticity in OXYS rats during active progression of Alzheimer’s disease-like pathology. J. Pineal Res..

[10] Korbolina E.E., Zhdankina A.A., Fursova A.Z., Kozhevnikova O.S., Kolosova N.G. (2016). Genes of susceptibility to early neurodegenerative changes in the rat retina and brain: Analysis by means of congenic strains. BMC Genet..

[11] Devyatkin V.A., Redina O.E., Kolosova N.G., Muraleva N.A. (2020). Single-Nucleotide Polymorphisms Associated with the Senescence-Accelerated Phenotype of OXYS Rats: A Focus on Alzheimer’s Disease-Like and Age-Related-Macular-Degeneration-Like Pathologies. J. Alzheimers Dis..

[12] Devyatkin V.A., Muraleva N.A., Kolosova N.G. (2019). Assessment of Mitochondria-Associated Genes for Single Nucleotide Polymorphisms Linked to the Development of Hypertrophic Cardiomyopathy in the Senescence-Accelerated OXYS Rats. Adv. Gerontol..

[13] Ng P.C., Henikoff S. (2006). Predicting the effects of amino acid substitutions on protein function. Annu. Rev. Genom. Hum. Genet..

[14] Kinoshita K., Ashenagar M.S., Tabuchi M., Higashino H. (2011). Whole rat DNA array survey for candidate genes related to hypertension in kidneys from three spontaneously hypertensive rat substrains at two stages of age and with hypotensive induction caused by hydralazine hydrochloride. Exp. Ther. Med..

[15] Stoll M., Kwitek-Black A.E., Cowley A.W., Harris E.L., Harrap S.B., Krieger J.E., Printz M.P., Provoost A.P., Sassard J., Jacob H.J. (2000). New target regions for human hypertension via comparative genomics. Genome Res..

[16] Kato N., Nabika T., Liang Y.Q., Mashimo T., Inomata H., Watanabe T., Yanai K., Yamori Y., Yazaki Y., Sasazuki T. (2003). Isolation of a chromosome 1 region affecting blood pressure and vascular disease traits in the stroke-prone rat model. Hypertension.

[17] Kloting I., Kovács P., Van den Brandt J. (2001). Quantitative trait loci for body weight, blood pressure, blood glucose, and serum lipids: Linkage analysis with wild rats (*Rattus norvegicus*). Biochem. Biophys. Res. Commun..

[18] Pravenec M., Kren V., Krenová D., Zídek V., Simáková M., Musilová A., Vorlícek J., Lezin E.S., Kurtz T.W. (2003). Genetic isolation of quantitative trait loci for blood pressure development and renal mass on chromosome 5 in the spontaneously hypertensive rat. Physiol. Res..

[19] Thiri M., Honda K., Kashiwase K., Mabuchi A., Suzuki H., Watanabe K., Nakayama M., Watanabe T., Doi K., Tokunaga K. (2016). High-density Association Mapping and Interaction Analysis of PLA2R1 and HLA Regions with Idiopathic Membranous Nephropathy in Japanese. Sci. Rep..

[20] Troyanov S., Wall C.A., Miller J.A., Scholey J.W., Cattran D.C., Toronto Glomerulonephritis Registry Group (2004). Idiopathic membranous nephropathy: Definition and relevance of a partial remission. Kidney Int..

[21] Pocivalnik M., Tsybrovskyy O., Schwarz C., Rosenkranz A.R., Eller K., Eller P. (2013). Role of anti-phospholipase A(2) receptor antibodies in the differential diagnosis of diabetic and membranous nephropathy. Diabetes Care.

[22] Griveau A., Wiel C., Le Calvé B., Ziegler D.V., Djebali S., Warnier M., Martin N., Marvel J., Vindrieux D., Bergo M.O. (2018). Targeting the phospholipase A2 receptor ameliorates premature aging phenotypes. Aging Cell.

[23] Cardenas-Rodriguez M., Irigoín F., Osborn D.P., Gascue C., Katsanis N., Beales P.L., Badano J.L. (2013). The Bardet-Biedl syndrome-related protein CCDC28B modulates mTORC2 function and interacts with SIN1 to control cilia length independently of the mTOR complex. Hum. Mol. Genet..

[24] Novas R., Cardenas-Rodriguez M., Lepanto P., Fabregat M., Rodao M., Fariello M.I., Ramos M., Davison C., Casanova G., Alfaya L. (2018). Kinesin 1 regulates cilia length through an interaction with the Bardet-Biedl syndrome related protein CCDC28B. Sci. Rep..

[25] Croft J.B., Swift M. (1990). Obesity, hypertension, and renal disease in relatives of Bardet-Biedl syndrome sibs. Am. J. Med. Genet..

[26] Elbedour K., Zucker N., Zalzstein E., Barki Y., Carmi R. (1994). Cardiac abnormalities in the Bardet-Biedl syndrome: Echocardiographic studies of 22 patients. Am. J. Med. Genet..

[27] Danckwardt S., Hentze M.W., Kulozik A.E. (2008). 3′ end mRNA processing: Molecular mechanisms and implications for health and disease. EMBO J..

[28] Greenbaum L., Smith R.C., Rigbi A., Strous R., Teltsh O., Kanyas K., Korner M., Lancet D., Ben-Asher E., Lerer B. (2009). Further evidence for association of the RGS2 gene with antipsychotic-induced parkinsonism: Protective role of a functional polymorphism in the 3′-untranslated region. Pharm. J..

[29] Mendelova A., Holubekova V., Grendar M., Zubor P., Svecova I., Loderer D., Snahnicanova Z., Biringer K., Danko J., Lasabova Z. (2018). Association between 3′UTR polymorphisms in genes ACVR2A AGTR1 and RGS2 and preeclampsia. Gen. Physiol. Biophys..

[30] Saxena A., Moshynska O., Sankaran K., Viswanathan S., Sheridan D.P. (2002). Association of a novel single nucleotide polymorphism G(-248)A in the 5′-UTR of BAX gene in chronic lymphocytic leukemia with disease progression and treatment resistance. Cancer Lett..

[31] Han Y.J., Ma S.F., Wade M.S., Flores C., Garcia J.G. (2012). An intronic MYLK variant associated with inflammatory lung disease regulates promoter activity of the smooth muscle myosin light chain kinase isoform. J. Mol. Med. (Berl.).

[32] Hong M.J., Lee S.Y., Choi J.E., Kang H.G., Do S.K., Lee J.H., Yoo S.S., Lee E.B., Seok Y., Cho S. (2018). Intronic variant of EGFR is associated with GBAS expression and survival outcome of early-stage non-small cell lung cancer. Thorac. Cancer.

[33] Okamoto K., Hazama F., Yamori Y., Haebara H., Nagaoka A. (1975). Pathogenesis and prevention of stroke in spontaneously hypertensive rats. Clin. Sci. Mol. Med..

[34] Henning E.C., Warach S., Spatz M. (2010). Hypertension-induced vascular remodeling contributes to reduced cerebral perfusion and the development of spontaneous stroke in aged SHRSP rats. J. Cereb. Blood Flow Metab..

[35] Stefanova N.A., Maksimova K.Y., Rudnitskaya E.A., Muraleva N.A., Kolosova N.G. (2018). Association of cerebrovascular dysfunction with the development of Alzheimer’s disease-like pathology in OXYS rats. BMC Genom..

[36] Markel A.L., Shishkina G.T. (1992). Genetic correlation between the arterial pressure response during emotional stress and the alpha1-adrenoreceptor concentration in brain regions. Genetika.

[37] Shmerling M.D., Buzueva I.I., Korostyshevskaia I.M., Lazarev V.A., Maksimov V.F., Filiushina E.E., Markel’ A.L., Iakobson G.S. (2005). Stereomorphometric Study of Target Organs in Rats With Hereditary Stress-Induced Arterial Hypertension at Different Periods of Postnatal Ontogenesis Under Changed Conditions of Nursing. Morfologiia.

[38] Muraleva N.A., Devyatkin V.A., Kolosova N.G. (2017). Phosphorylation of αB-crystallin in the myocardium: Analysis of relations with aging and cardiomyopathy. Exp. Gerontol..

[39] Ragaeva D.S., Tikhonova M.A., Petrova O.M., Igonina T.N., Rozkova I.N., Brusentsev E.Y., Amstislavskaya T.G., Amstislavsky S.Y. (2017). Neonatal reflexes and behavior in hypertensive rats of ISIAH strain. Physiol. Behav..

[40] Hermsen R., De Ligt J., Spee W., Blokzijl F., Schäfer S., Adami E., Boymans S., Flink S., Van Boxtel R., Van der Weide R.H. (2005). Genomic landscape of rat strain and substrain variation. BMC Genom..

[41] Zheng X., Levine D., Shen J., Gogarten S.M., Laurie C., Weir B.S. (2012). A high-performance computing toolset for relatedness and principal component analysis of SNP data. Bioinformatics.

